# Medical students' and doctors' attitudes towards older patients and their care in hospital settings: a conceptualisation

**DOI:** 10.1093/ageing/afv082

**Published:** 2015-07-15

**Authors:** Rajvinder Samra, Amanda Griffiths, Tom Cox, Simon Conroy, Adam Gordon, John R. F. Gladman

**Affiliations:** 1 Department of Primary Care and Public Health, Imperial College London, London, UK; 2 Division of Psychiatry and Applied Psychology, University of Nottingham, School of Medicine, Nottingham, UK; 3 Centre for Sustainable Working Life, School of Business, Economics & Informatics, Birkbeck University of London, London, UK; 4 University Hospitals of Leicester, Leicester, UK; 5 Division of Rehabilitation and Ageing, University of Nottingham, School of Medicine, Nottingham, UK

**Keywords:** physicians, attitudes, older patients, interview study, UK, older people

## Abstract

**Background**: despite assertions in reports from governmental and charitable bodies that negative staff attitudes towards older patients may contribute to inequitable healthcare provision for older patients when compared with younger patients (those aged under 65 years), the research literature does not describe these attitudes in any detail.

**Objective**: this study explored and conceptualised attitudes towards older patients using in-depth interviews.

**Methods**: twenty-five semi-structured interviews with medical students and hospital-based doctors in a UK acute teaching hospital were conducted. Participants were asked about their beliefs, emotions and behavioural tendencies towards older patients, in line with the psychological literature on the definition of attitudes (affective, cognitive and behavioural information). Data were analysed thematically.

**Results**: attitudes towards older patients and their care could be conceptualised under the headings: (i) beliefs about older patients; (ii) older patients' unique needs and the skills required to care for them and (iii) emotions and satisfaction with caring for older patients.

**Conclusions**: our findings outlined common beliefs and stereotypes specific to older patients, as opposed to older people in general. Older patients had unique needs concerning their healthcare. Participants typically described negative emotions about caring for older patients, but the sources of dissatisfaction largely related to the organisational setting and system in which the care is delivered to these patients. This study marks one of the first in-depth attempts to explore attitudes towards older patients in UK hospital settings.

## Introduction

Hospitals struggle to meet demand for acute care, because they are unprepared and unequipped for older patients [1]: this may be partly due to ‘underlying and widespread ageism’ [2]. Studies of medical students and doctors have mainly used questionnaires and have often examined attitudes towards older people in general rather than as patients [3–6], yet two American qualitative studies exploring doctors' attitudes [7, 8] found them to be specific to patients rather than simply older people – for example, that older patients can be more satisfying to treat but are typically more challenging to manage. There has been very little research about medical students' or doctors' attitudes towards older patients which originates from the UK [9] and no UK qualitative studies on the topic. We aimed to explore medical students' and doctors' attitudes towards older patients in the UK and chose to advance understanding by guiding our research using the theoretical approach to attitudes that defines them as judgements based on stereotypes/cognitions (beliefs about older patients), affective information (emotions and feelings experienced when dealing with older patients) and behavioural-related information (past and future behavioural intentions in relation to older patients) [10].

## Method

### Theoretical background

The present research is exploratory and theoretically underpinned by a post-positivist perspective [11]. Post-positivists [11–13] argue that because knowledge is based on human conjecture [12], we can only know reality imperfectly and probabilistically [11]. From this perspective, qualitative research allows the study of an additional dimension of knowledge (such as personal experience and meaning) [13, 14] to quantitative research, allowing us to approximate truth with more probability, similar to the process of data triangulation [14].

### Design

In-depth, semi-structured individual interviews were used. An interview guide was used to allow the interviewer to focus on what is being said [15]. The flexibility of a semi-structured format allows the interviewer to pose questions not on the interview guide if they are deemed relevant to the research [16].

### Setting and participants

Participants were recruited from a large teaching hospital in the English Midlands. Participants were medical students or doctors. All doctors apart from paediatricians were eligible to participate. Medical student participants were in their clinical years to ensure that they had experience of hospitals and interacting with patients. Participants were accessed through advertisements on hospital noticeboards and via email lists. Where an initial low response was identified in particular groups (surgeons, junior doctors and consultants), two consultants working at the hospital were asked to support recruitment. Twenty-five participants were recruited, of which 15 (60%) were female*.* Interviewing ended when no new themes appeared to be presented during interviews. Each participant was assigned a number that accompanies their quotes presented in the findings. Characteristics of the sample are presented in Table 1.

**Table 1.** Demographic characteristics of participants

Participant ID	Gender	Age range	Role	Level/grade	Specialty	Years of practice
P1	Female	21–30	Medical student	Year 5	–	–
P2	Female	21–30	Medical student	Year 4	–	–
P3	Female	21–30	Medical student	Year 4	–	–
P4	Male	41–49	Doctor	Consultant	Geriatric medicine	26
P5	Female	21–30	Medical student	Year 5	–	–
P6	Male	41–50	Doctor	Consultant	Geriatric and stroke medicine	25
P7	Female	21–30	Doctor	Specialty trainee registrar	Geriatric medicine	6
P8	Male	21–30	Medical student	Year 4	–	–
P9	Female	31–40	Doctor	Specialty registrar	Geriatric medicine	10
P10	Male	21–30	Medical student	Year 4	–	–
P11	Male	41–50	Doctor	Consultant	Stroke medicine	18
P12	Male	40–50	Doctor	Consultant	Diabetes and endocrinology	14
P13	Female	40–50	Doctor	Consultant	Stroke medicine	19
P14	Female	21–30	Medical student	Year 4	–	–
P15	Female	21–30	Doctor	Foundation Year 1	–	∼1
P16	Female	21–30	Doctor	Foundation Year 2	–	∼2
P17	Male	21–30	Doctor	Specialty trainee registrar	Plastics and reconstructive surgery	5
P18	Female	21–30	Doctor	Foundation Year 2	–	∼2
P19	Female	21–30	Doctor	Foundation Year 2	–	∼2
P20	Female	21–30	Doctor	Foundation Year 2	–	∼1
P21	Male	31–40	Doctor	Consultant	Respiratory medicine	13
P22	Male	31–40	Doctor	Consultant	Geriatric medicine	10
P23	Female	41–50	Doctor	Consultant	Acute medicine	16
P24	Female	21–30	Doctor	Foundation Year 1	–	∼1
P25	Male	51–60	Doctor	Consultant	General surgery	29

Dash indicates ‘data not applicable’.

### Procedure

All interviews took place in a private space at the hospital between September and October 2011. Participants were given an information sheet and asked to sign a consent form. Interviews were conducted by one researcher, were digitally recorded lasting an average of 50 min and professionally transcribed. Ethical and regulatory approvals were obtained (REC 10/H0408/114).

### Developing the interview guide

The areas of inquiry were informed by our chosen definition of attitudes [10]: characteristics of older patients and their care (cognitions/stereotypes); descriptions of how interactions with and behaviour towards older patients may differ from other patient groups (behavioural tendencies); and emotions experienced when dealing with older patients and their care (affective information). Potential questions went through multiple iterations to minimise ambiguity of wording. Two researchers formulated the interview schedule. Attempts were made to ensure that question phrasing was neutral and not value-laden [15]. A funnelling technique was used to sequence questions, reducing the risk of bias in the direction of the interviewer's views. In this, general questions are asked at the beginning, followed by more specific questions where appropriate. The interview guide was piloted on one participant whose results were not included in the analysis. Example questions from the interview schedule are included below (see the Supplementary data, Appendix, available in *Age and Ageing* online for full interview guide).

- *Questions about stereotypes and beliefs*: Do members of the older patient population tend to differ from younger patients?
- *Questions about behavioural tendencies*: Do you feel you need to act, or behave, differently towards older patients to deliver good care?
- *Questions about affect*: Can you describe something you find enjoyable about working with older patients?

### Data analyses

Thematic analysis was conducted [17] using inductive coding organised using NVivo qualitative data analysis software. Transcripts were checked against audio recordings for accuracy and read multiple times for familiarity. Initial coding and analysis was conducted by one researcher. Initial coding attached basic surface-level labels to data, resulting in ∼600 codes. Basic codes addressing similar phenomena were then collapsed to form categories. Categories were grouped into clusters according to shared characteristics (subthemes). Clusters were then analysed for larger themes. Four researchers commented separately on the relationship between themes and subthemes. The theme development stage closed when consensus was reached. The themes, subthemes and categories, along with the anonymised coded transcripts, were given to the second coder for inspection and comments. Differences in interpretations were raised, discussed and resolved when consensus was reached between the two coders.

## Findings

Findings were organised into three themes: (i) beliefs about older patients, (ii) older patients' unique needs and the skills required and (iii) emotions and satisfaction related to caring for older patients (Table 2).

**Table 2.** Attitudes towards older patients and their care: themes, subthemes and categories

Theme	Subtheme	Category
Beliefs about older patients	Composure and manner	Being respectful and polite
		Demonstrating gratitude
		Demonstrating trust
		Demonstrating resilience in adversity
		Displaying hostile or challenging behaviours
	Communication skills	Being conversational
		Limited by level of cognitive impairments
		Affected by memory issues
		Affected by limitations in information processing
		Affected by sensory impairments
	Biological age	Physical limitations
		Chronological versus functional age
	Heightened vulnerability in hospital	Isolation and loneliness
		Distress
		Fragility and risk
Older patients' unique needs and the skills required from doctors and medical students	Taking complex patient histories	Increased importance of the history
		Accessing and corroborating information from others
		Time-consuming and longer histories
	The challenge of diagnosis	Multimorbidity, co-morbidity and multiple medications
		Atypical presentations and non-specific symptoms
		Potential for misdiagnoses and missed diagnoses
		Constraints of performing thorough examinations
	Communication with patients and relatives	Need for clarity and brevity of speech
		Being patient with the patient
		Reassuring the patient
		Managing paternalistic tendencies in self and relatives
	Determining the treatment plan	Appropriate level of treatment
		Negotiating with relatives and others about treatment
		Prioritising illnesses to deal with patient complexity
		Importance of treating the whole person
		Preventing complications or worsening of patient health
		Problem of ‘social admissions’
	Organising a safe discharge and future rehabilitation needs	Necessity of multidisciplinary teams for safe discharge
		Challenge of achieving a timely discharge
	Becoming a good doctor to older patients	Under-representation of older patient care issues in medical curricula
		Developing compassion and patience
		Whether dealing with complexity is a teachable skill
		Learning to hone in on the most important aspects of illness
		Changing training focus and performance standards for medics
		Importance of teachers and senior doctors
Emotions and satisfaction related to caring for older patients	Fear and anxiety	Not doing enough or knowing enough
		Anxiety about interacting with patients
	Sadness and compassion	Experiencing patient deterioration and death
		Witnessing patient loneliness
		Thinking about the person behind the illness
	An imperfect system for older patients	Underserving older people
		Exclusion in policy and research
		Extended wait for discharge
		Staffing levels
		Bed pressures
		Time and efficiency pressures
	Dealing with a highly complex patient group	Dealing with communication difficulties
		Perceived mismanagement of care
		Less opportunity to cure patients
		Intellectual challenge
		Variety of work
		Working as a team
	Improving the patients' quality of life	Getting the patient out of hospital safely
		Supporting the patient and their family
		Providing a good death
		Social justice

### Beliefs about older patients

#### Composure and manner

Older patients were described as polite and respectful towards doctors and medical students. They often expressed gratitude for medical care and placed great trust in doctors and medical students. They were often described as resilient and tolerant: (*‘*They'll quite happily sit there and say “oh no I don't mind waiting a little bit longer, oh no I'm not in pain, don't worry”’, P7). The delivery of bad news, such as terminal prognoses, to older patients was frequently less difficult as they seemed better prepared for it. A few participants described hostile and aggressive behaviours in older patients but exclusively reported these in conjunction with dementia or delirium.

#### Communication skills

Older patients were described as talkative and interesting. A number of participants speculated that this desire for interaction was a result of social isolation at home. A few participants described the ability of older patients to engage in two-way conversation with doctors even when they had cognitive impairment, which could mask their cognitive impairment. For patients with cognitive impairment, communication skills were highly variable, both between patients and within the same patient over time. Communication problems in cognitive impairment posed diagnostic and management difficulties: ([Sometimes] *‘*they're non-communicative or they are very confused or they don't tend to talk in full sentences or they answer inappropriately’*,* P18). Older patients were more likely to present with confusion as an accompaniment to physical illness which meant that they may not recall important clinically relevant events such as falls. Some participants suggested that older patients with more severe memory impairment may not retain instructions or recall recent conversations with doctors.

#### Biological age

Participants reported physical limitations, such as impaired mobility, that affected older patients' level of activity in hospital and life immediately after hospital admission. Participants distinguished between patients' ‘functional age’ (i.e. equating greater functional age to greater physical and mental functional limitations) and their ‘chronological age’. Commonly, participants reported that functional age carried more weight than chronological age when managing older patients:

… being old isn't really a numerical value, I think the idea of being old is perhaps how a patient looks… how mobile they are, how sort of independent they are, how much that they can do, I don't think it's as simple as putting a number on things (P5).

#### Heightened vulnerability in hospital

Participants described how older patients frequently appear to be isolated, both at home and when they came into hospital: ‘you might be the first person they have had a proper talk with for weeks or months even*’* (P20). Multiple participants described the sadness that some older patients appeared to experience when discharged. A few participants suggested that the hospital environment can be frightening, magnified by symptoms of illness, such as confusion: ‘…[older] patients are often scared silly and they never remember anything because they're so anxious’ (P4). Older patients in hospital were generally reported to have fragile health, reduced immunity and longer recovery times following illness. A few participants made clear that older patients sometimes suffered longer standing health impairment as a consequence of a hospital admission.

### Older patients' unique needs and the skills required from doctors and medical students

#### Taking complex patient histories

Taking a good history was described as important in older patients due to increased likelihood of co-existing illness and polypharmacy, but it was commonly described as challenging and time-consuming. Participants commonly described the ‘social history’ as crucial to developing a picture of older patients and their normal functioning and that such insights could help avoid putting patients at risk of harm: ‘it doesn't matter if you cure their pneumonia, if you're going to send them home and they can't wash and feed themselves, then they're going to starve to death’ (P16). There was a need to cross-check information, because patients may have forgotten aspects of their history. This collateral history-taking from carers or relatives was not always straightforward, because they did not always know or remember patients' medical and drug history. Collateral history-taking could include contacting staff at care homes or GP surgeries, which could be difficult when patients were admitted outside of traditional working hours:

I've had a case whereby he couldn't actually confirm the drug history for a few days… obviously it's a danger for patients who are on insulin and things like that. You'd have to start kind of guestimating exactly what you need to give them (P24).

#### The challenge of diagnosis

The frequent occurrence of having to diagnose patients based on vague and non-specific symptoms was challenging: ‘So they may just present with a fall and having followed them and then you examine them and you find they've got pneumonia’ (P6). Multiple illnesses and multiple medications meant that doctors may find multiple underlying issues and not a single diagnosis. Participants reported greater opportunity for misdiagnosis and for missed diagnoses, such as mistaking depression for dementia or confusion in withdrawn and unresponsive patients.

#### Communication with patients and relatives

There was often a need to adjust communication style for older patients. Typically, participants reported needing time to communicate: to speak slower and louder without jargon and repeat information, when necessary, for older patients to process questions and formulate responses. Participants claimed that it can be easy to just move on with management without fully checking what cognitive or hearing impaired patients understand or want from their care. Paternalism also needed to be managed. Participants reported that older patients sometimes wanted them to take on a paternalistic role in treatment and care. They also reported they might pay more attention to relatives or carers than the patients themselves, which they were aware might be paternalistic: ‘The families are perhaps… a bit more realistic about what might be good for the patient than the patient themselves’ (P20).

#### Determining the treatment plan

Being able to identify what to treat and not to treat was a frequently identified concern. Multiple participants explained that getting older patients back to previous levels of functioning was the main goal of treatment, because curing all health problems was unlikely. Offering less treatment or less aggressive treatment might be more appropriate than putting older patients through invasive tests or prescribing multiple medications. With complex older patients, the doctor may need to identify all the possible factors underlying the admission and then prioritise each one: ‘make a problem list… make it manageable and make it sort of bite size as it were*’* (P7). The prevention of secondary problems and complications was a crucial part of treatment. Participants reported the need for accuracy and caution when treating older patients as they typically have less physiological ability to compensate should something go wrong. Avoidable complications need to be managed:

… but I mean things I'm worried about are pressure areas, UTIs [Urinary Tract Infections], chest infections, aspiration, immobility, DVTs [Deep Vein Thrombosis], sundowning, confusion and all those sorts of things (P21).

Sometimes, there was a need to negotiate the treatment plan with other doctors who expected less active treatment for older patients, because they saw the illness as a consequence of ageing: ‘…especially amongst surgeons, there's a big view that “they're just old”, you know, “we are not going to do anything because they are old”’ (P18). Participants differed on the effect of an entry of ‘social admission’ on the treatment plan. One perspective suggested a ‘social admission’ is just a hospital stay to ensure that the older patient is safe, because they ‘cannot cope’ and that ‘nothing is medically wrong with them’ (P23). The other perspective saw this reasoning as ageist as it excuses medical inaction, when in fact a number of medical things have indeed gone wrong.

#### Organising a safe discharge and future rehabilitation needs

Caring for older patients was described as reliant on multidisciplinary working. Team decision-making was used to ensure discharge decisions were well-reasoned and safe. One of the most difficult parts of caring for older patients' was achieving a timely discharge from hospital, which was described as protracted and difficult. Older patients typically required more assistance when they got home, and they were often kept in hospital until the medical team was confident that their home care needs would be met. Some participants reported that older patients could wait over a month for a care package to be organised, and that this wait itself could threaten patients' health.

#### Becoming a good doctor to older patients

Participants reported that their medical education had not provided them with realistic information about the proportion of older patients in hospital or developed the necessary skills to be a good doctor to older patients. They explained that the curriculum focussed on patients with single illnesses which may be cured entirely. Some participants, mostly consultant doctors from various specialties, stated that the skills needed to treat older patients cannot be taught to undergraduates but had to be learnt on the job: undergraduate education was seen only to teach students' theoretical prototypes of illness. However, one consultant doctor claimed dealing with complexity to be a specific skillset which could be taught by the right educators but is not taught well at present. He claimed that doctors competent in dealing with complexity should have to communicate the steps they take and illustrate the logic that underpins their decision-making in older patient care decisions. There was a tendency for participants, regardless of seniority or specialty, to describe the influence senior doctors had on them. Role models or inspirational teachers appeared to be very important to junior doctors, because the ability to deliver good care is learned from watching senior doctors. Across the different medical specialities, participants reported being interested in a particular specialty but changing their mind because they did not identify a suitable role model. Role models were described as: ‘someone who wants what you want out of life and has found it in that profession, and therefore, you can see it's possible’ (P19).

### Emotions and satisfaction related to caring for older patients

#### Fear and anxiety

Participants predominantly described negative emotions about working with older patients. Medical students and junior doctors provided examples of feeling anxious when interacting with older patients. Junior doctors described feeling self-doubt, anxiety and guilt when older patients' health deteriorated. Decline and death sometimes led to participants' questioning whether they had done enough for the patient. Typically, these feelings of self-doubt were only described by junior doctors. A few of the specialists in geriatric or stroke medicine explained how junior doctors sometimes appeared uncomfortable with older patients because of an underlying fear of making errors which manifested in ‘abhorrent reactions from them like, “why bother, they're going to die anyway, they're at the end of their life, it's a hopeless case, there's nothing I can do”’ (P22).

#### Sadness and compassion

Participants commonly described sadness when caring for older patients, and this was often linked to witnessing an older patient's deterioration or death. This was sometimes accompanied by remarks about feelings of helplessness and hopelessness for older patients and their health. Sadness at witnessing decline in health was not often mentioned by those more experienced in hospital medicine. A more senior doctor described how, with experience, the feelings of sadness diminish, because doctors become more knowledgeable and realistic about what can be achieved: ‘… with experience you think really there's only so much you can do. You can cure people, you can palliate people but you can't prevent death’ (P12). Some participants found caring for older patients saddening, because they often appeared to be lonely and lack family support. Remembering that an older patient was of a similar age to a parent or grandparent made some feel more compassionate:

A lot of the people you know are elderly, your parents are starting to get older, you may think back to your grandparents, you may even look at yourself and go ‘I'm going to be elderly one day’ (P3).

#### Frustration with the imperfect system for older patients

Some participants described what they thought was a dominant societal belief: that effort, time and money spent on younger people were considered to be more worthwhile. Consultant doctors commented on the lack of medical research involving older patients and a general disinterest in involving older patients in health service design and delivery. A consultant surgeon explained his dissatisfaction that treatments are researched on younger populations which may be inappropriate as cancer can be different in older people. Bed utilisation and the extended wait for safe discharge was one of the most commonly mentioned sources of frustration and dissatisfaction, often blamed upon the social services. Many participants reported dissatisfaction, because time and efficiency pressures meant that they could not provide the standard of care for older patients that they would like to.

#### Dealing with a highly complex patient group

Some participants described dissatisfaction at mismanagement of older patients by other doctors, such as after surgery on an orthopaedic ward or by GPs. Another important source of dissatisfaction related to ‘social admissions’: ‘I think that patients that get these labels attached to them often do end up receiving second-rate care’ (P22). It was also common for medical students and junior doctors to report that they did not get as much satisfaction from helping older patients get better in comparison with younger patients because it was ‘fighting the inevitable’ (P10) or because some keep coming back to hospital—‘revolving door’ (P18) patients. In stark contrast, doctors of a more senior grade reported that getting older patients back to their best health is akin to curing. Doctors working in specialties with many complex older patients described feeling satisfied by the intellectual challenge of solving complex problems that commonly present in this group: ‘My job is never boring… there is no way in which I am a technician or a glorified technician… I like the analytical work in unravelling problems’ (P4). In contrast, those working in settings where older patients presented less frequently (such as surgical specialties) tended to remark on the unvaried and boring nature of the work: ‘I'm just like, “Oh no, another old person with a fall”’ (P16).

#### Improving older patients' quality of life

Supporting older patients and their families was reported as involving both elements of satisfaction and dissatisfaction. A common source of satisfaction when treating older patients was the feeling that the participant had helped to ensure older patients remained safe during admission and after discharge. Participants sometimes supported cognitively impaired older patients' relatives rather than the patient themselves: some reported this as rewarding but others described the difficulty of dealing with older patients' relatives, especially those who had unrealistic expectations. Providing ‘a good death’ for older patients was a source of satisfaction and a number of participants reported how this was completely unexpected and could be ‘life-affirming’ (P21) for doctors. Geriatricians, specifically, also described the satisfaction and a sense of social justice experienced by working in an unpopular but needed medical specialty.

## Discussion

This study found that the attitudes of medical students and doctors towards older patients and their care are multi-dimensional and consider characteristics of older patients, the typical healthcare processes involved, and the personal and organisational environment of the individual providing their care. Beliefs about older patients included seeing them as respectful towards medical staff, the notion of the importance of ‘functional age’ and their vulnerability. The behavioural aspects of their attitudes concerned communication problems affecting diagnosis and management, particularly with patients with cognitive impairment. Overall, emotional responses were negative, although this was mainly in more junior staff.

The qualitative nature of this study was more illuminating than a closed-item questionnaire study might have been. Participants chosen were carefully sampled to draw from a range of medical participants and levels of seniority, enabling diversity to be seen that would not be apparent with a more narrow selection process. Despite achieving saturation, this study was small, and it is not known whether the attitudes described here are reflective of any wider group, and attitudes were likely to be affected by job role and length of working experience of participants. The findings were not subject to a validity check with members of the participant group, as many participants moved away from the study hospital, but this is unlikely to invalidate the findings.

This study did not identify general stereotypes that related to simply being an older person: attitudes were strongly shaped by the context of illness experienced in hospital. General older person-targeted attitude questionnaires typically include items irrelevant to the healthcare context such as ‘older people have too much power in business and politics’ [18]. Future research pertaining to attitudes towards older patients to explore possible ageism in health care should use information specific to the health-related context and use older patients rather than older people as the target of investigation. Future work could also investigate how social and cultural beliefs about ageing infuse with the healthcare professional's attitudes towards older patients.

Many of the negative emotional attitudes particularly reported by junior staff related to inadequacies in their training, the healthcare system and the organisational context of care. Most participants described an imperfect system for providing good quality care, related to poor training, low staffing levels, time and efficiency pressures, and bed pressures. Affective information (emotions, satisfaction or dissatisfaction) was often described in relation to the provision of care for the older patient, rather than the characteristics of the older patients themselves. Our findings echo those of Tadd *et al*. [2] wherein staff attitudes towards providing care to older patients were shaped by the organisational and social context of the acute trust hospital, resulting in attitudes that older patients were ill-suited to the environment rather than the environment is ill-equipped to deal with its largest patient group. Thus, these findings indicate ways that some of the negative attitudes towards older patients might be improved, such as through more realistic undergraduate education, and in services that are adequately resourced, managed and led.

These findings, in particular the areas that led to satisfaction also might help doctors in training to identify the care of older people as a rewarding area of work and help those with a responsibility to develop a workforce of the future fit to manage ageing populations. Such areas included seeing vulnerable people through complex episodes of care, the intellectual challenges of complex care and witnessing good deaths.

These findings provide a more nuanced picture than of ‘underlying and widespread ageism’ [2] among medical staff in the UK hospital setting. Further work is needed to explore these attitudes in other groups and locations, and to develop quantitative tools to enable even more systematic examination of the causes and consequences of medical students' and doctors' attitudes towards older patients.

**Key points**

- Explored medical students' and doctors' attitudes towards older patients and their care.
- Attitudes were multi-dimensional and complex.
- Older patients' had unique needs concerning their health care.
- Respondents described mostly negative emotions about caring for older patients.
- Sources of dissatisfaction involved the organisational setting and system.

## Conflicts of interest

None declared.

## Funding

The study was funded by an Economic and Social Research Council Studentship award (ES/H014659/1) in the corresponding author's name. The funding source had no involvement in the collection, analysis and interpretation of data; in the writing of the report and in the decision to submit the article for publication. The corresponding author had full access to the data.

## Ethical approval

The study was approved by a NHS Research Ethics Committee (10/H0408/114) and approved by the local Research and Development department at the NHS Trust. All participants gave informed consent.

## Supplementary data

Supplementary data mentioned in the text are available to subscribers in *Age and Ageing* online.

Supplementary Data
