# Supplementary material for: Medical students' and doctors' attitudes towards older patients and their care in hospital settings: a conceptualisation
Source: Age Ageing. 2015 Jul 15;44(5):776–83. doi: 10.1093/ageing/afv082 (PMC4547928; doi:10.1093/ageing/afv082)
Supplement: Supplementary Data [file supp_44_5_776__index.html]

Medical students' and doctors' attitudes towards older patients and their care in hospital settings: a conceptualisation — Supplementary Data 

# Medical students' and doctors' attitudes towards older patients and their care in hospital settings: a conceptualisation

## Supplementary Data

Supplementary Data

- Supplementary Data - Docx file
