# Supplementary material for: Medical students' and doctors' attitudes towards older patients and their care in hospital settings: a conceptualisation
Source: Age Ageing. 2015 Jul 15;44(5):776–83. doi: 10.1093/ageing/afv082 (PMC4547928; doi:10.1093/ageing/afv082)
Supplement: Supplementary Data [file supp_afv082_afv082supp.docx]

*SUPPLEMENTARY DATA*

*Appendix: Interview guide*

Background:

1. Roughly, how long ago did you begin your experience on the hospital wards?
2. If I asked you at what age a patient should be deemed elderly or an “older patient”, what kind of age would you be thinking of?
3. How often do interact with older patients at work (65 years or over)?
4. On the general wards, can you roughly describe what kind of proportion of older patients make up the patient group you encounter?

For the rest of this interview, when I refer to ‘older patients’, I will mean those over the age of 65 years.

Questions about stereotypes and beliefs

1. Do older patients differ from younger patients in any ways?
2. (i) Do you think older patients have different care needs to other patients?

(ii) If so, can you describe the differences?

Questions about behaviour tendencies

1. (i) Do you feel you need to act, or behave, differently toward older patients to deliver good care? … If so, can you describe some examples?
2. (i) Are there any differences in the way that you have to interact with older patients compared to other patients?...Can you describe any recent examples of a time when you had to interact differently with an older patient?
3. Do you see differences between doctors from different specialities in how they interact and deal with older patients?...In what ways?
4. (i) Have you noticed any ways in which you have changed how you deal with older patients as you have gained more experience on the wards?
5. (ii) Have you noticed changes in others that you work with, in how they deal with older patients as they gain experience?

Questions about affect

1. Can you describe something you find enjoyable about working with older patients?
2. Can you describe something you find challenging about working with older patients?
3. Are there any emotions you experience when dealing with older patients, that were unexpected or you were surprised by?

Demographics

1. May I have your age?
2. How would you describe your ethnic background?
